# Cross-cultural development of an EORTC questionnaire to assess health-related quality of life in patients with testicular cancer: the EORTC QLQ-TC26

**DOI:** 10.1007/s11136-012-0147-1

**Published:** 2012-03-10

**Authors:** Bernhard Holzner, Fabio Efficace, Umberto Basso, Colin D. Johnson, Neil K. Aaronson, Juan I. Arraras, Allan B. Smith, Edward Chow, Anne S. Oberguggenberger, Andrew Bottomley, Hannes Steiner, Luca Incrocci, Johannes M. Giesinger

**Affiliations:** 1 Department of Psychiatry and Psychotherapy, Innsbruck Medical University, Anichstr. 35, 6020 Innsbruck, Austria; 2 Health Outcomes Research Unit, Italian Group for Adult Hematologic Diseases (GIMEMA) Data Center, Rome, Italy; 3 Medical Oncology 1, Istituto Oncologico Veneto (IOV) IRCCS, Padua, Italy; 4 Italian Germ Cell Cancer Group (IGG), Padua, Italy; 5 University Surgical Unit, Southampton University Hospitals, University of Southampton, Tremona Road, Southampton, SO16 6YD UK; 6 Division of Psychosocial Research and Epidemiology, The Netherlands Cancer Institute, Plesmanlaan 121, 1066 CX Amsterdam, The Netherlands; 7 Medical Oncology Department, Hospital of Navarre, C/Irunlarrea 3, 31008 Navarre, Pamplona, Spain; 8 Psycho-Oncology Co-operative Research Group, School of Psychology, University of Sydney, Sydney, NSW 2006 Australia; 9 Department of Radiation Oncology, Toronto Sunnybrook Regional Cancer Centre, 2075, Bayview Avenue, Toronto, ON M4N 3M Canada; 10 EORTC Headquarters Quality of Life Department, Ave. E. Mounier 83, 11 1200 Brussels, Belgium; 11 Center of Operative Medicine, Innsbruck Medical University, Anichstr. 35, 6020 Innsbruck, Austria; 12 Department of Radiation Oncology, Erasmus MC-Daniel Den Hoed Cancer Center, Rotterdam, The Netherlands

**Keywords:** Testicular cancer, Quality of life, Questionnaire, eortc qlq-tc26

## Abstract

**Objective:**

Testicular cancer (TC) is the most common cancer in young men, and its incidence is increasing. The low mortality rate makes quality of life (QOL) an important issue in this patient group. This study aimed to develop a supplementary module of the EORTC QLQ-C30 questionnaire to assess TC-specific aspects of QOL.

**Methods:**

Questionnaire development was conducted according to guidelines from the EORTC Quality of Life Group. Phase I comprised generation of QOL issues relevant to TC patients through a literature search and interviews with patients and experts. Phase II included operationalization and assessment of item relevance. In phase III, items were pre-tested in a cross-cultural sample to assess issues such as understandability and intrusiveness of items.

**Results:**

In phase I and II, an initial list of 69 QOL issues possibly relevant to TC patients was refined through patient and expert interviews. The remaining 37 issues were operationalized into items and assessed for relevance and priority in an expert sample (*n* = 28) and a patient sample (*n* = 62) from Austria, Canada and the Netherlands. After revision of the item list, 26 items were considered eligible for pre-testing in phase III, in which 156 patients from Australia, Austria, Italy and Spain participated. All items passed criteria for pre-testing, thus forming the new EORTC QLQ-TC26.

**Conclusion:**

The newly developed EORTC QLQ-TC26 is now available in several languages to assess QOL in TC patients receiving treatment and in TC survivors. Phase IV of questionnaire development will comprise international field testing, including extensive analysis of psychometric characteristics of the EORTC QLQ-TC26.

## Introduction

Testicular cancer (TC) is the most common type of cancer in men aged 15–45 years, and its incidence is increasing [1, 2]. Due to the high survival rate in this cancer population, preserving quality of life (QOL) and minimizing adverse effects of cancer therapy are major issues [3–5]. These issues are particularly important as testicular cancer typically occurs as men are approaching the peak of their personal and professional lives, when fertility and family life are of utmost importance [6].

Treatment for TC usually comprises orchiectomy, with subsequent therapy depending on tumour histology and stage [7]. Patients with seminomas often receive additional radiotherapy [8], but also carboplatin-based chemotherapy has been shown to be a good alternative for stage I seminomas [9]. Chemotherapy with bleomycin, etoposide and cisplatin shows very good results for non-seminomas and seminomas with a stage higher than I [10]. Another therapy option in case of residual tumour mass after chemotherapy is retroperitoneal lymph node dissection with nerve sparing [2, 11].

To date, studies of symptom burden in TC patients have generally focused on survivors, highlighting a range of persistent impairments. Physical impairments relating to chemotherapy side effects include Raynaud’s phenomena [12, 13], tinnitus [12] and long-term effects such as increased incidence of cardiovascular disease [14, 15]. Both chemotherapy and radiotherapy impact infertility [16] and lead to increased fatigue levels [3].

TC survivors’ sexual functioning is impacted by gonadal dysfunction [17, 18], decreased libido [19], dry ejaculation [5, 19] and other sexual difficulties [20]. Fegg et al. [19] also argue that sexual concerns are further aggravated by inadequate communication about these issues between doctors and patients. Ozen et al. [21] contend that libido and erectile dysfunction improved post-treatment, but did not reach pre-treatment levels. In contrast, ejaculation problems increased further after cessation of treatment.

To date, a limited number of questionnaires have been specifically validated for use in TC patients, for example, to assess neurotoxicity [22], coping [23], marital and sexual satisfaction [24], and QOL [25]. In particular, the QOL questionnaire developed by Fossa et al. [25] was an important step towards the comprehensive assessment of symptoms and functioning in TC patients. This questionnaire has already been used to measure outcomes in an international trial of the European Organisation for Research and Treatment of Cancer (EORTC) investigating QOL in patients with metastatic germ cell cancer [12]. However, as mentioned by Fossa et al. [12], psychometric testing and extensive translation checks for this questionnaire have not been undertaken. Also, its development did not follow the detailed EORTC guidelines for questionnaire development guaranteeing cross-cultural applicability and compatibility with the EORTC QLQ-C30. The EORTC approach to QOL assessment is to use the EORTC QLQ-C30 for assessing general aspects of QOL that are relevant to (almost) all cancer patients and to supplement this core questionnaire with disease-specific questionnaire modules. So far, a questionnaire module for TC patients was lacking, limiting the use of the EORTC QOL measurement system with regard to this patient group.

Thus, the aim of this project was to develop a questionnaire module as a supplement to the EORTC QLQ-C30 to assess QOL of TC patients in clinical trials and daily clinical practice. This questionnaire module was designed to be applicable to both patients undergoing treatment and cancer survivors and covers TC-specific issues such as common treatment side effects, infertility, body image and sexuality.

## Methods

Our study followed the EORTC guidelines for developing questionnaire modules [26]. These guidelines comprise four phases: (1) generation of relevant QOL issues, (2) operationalization of the QOL issues into a set of items, (3) pre-testing the questionnaire module and (4) large-scale international field testing.

**Fig. 1:**
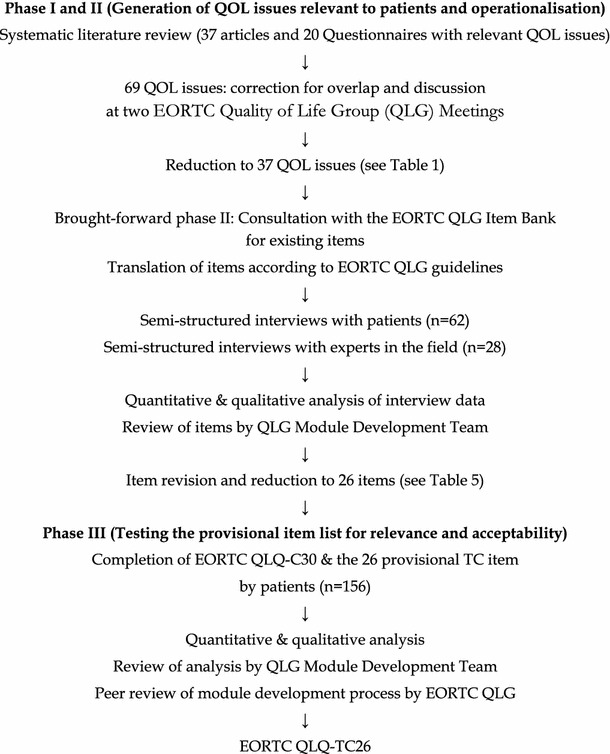
Flowchart of the module development process for the QLQ-TC26 (according to [26, 37])

Phase I-III has now been completed, and the results from each are presented within this manuscript. The main steps of the whole development process are summarized in Fig. 1.

### Phase I and II: Generation of relevant QOL issues and operationalization

An extensive literature search was conducted to establish an initial list of QOL issues potentially relevant to TC patients. This list was evaluated in semi-structured interviews with experts in the field and with patients to clarify whether further issues should be included.

The literature search in the databases MEDLINE and PsychINFO covered the years 1996 to 2006. The following keywords were used: (testis OR testicular) AND (carcinoma OR cancer OR neoplasm) AND (quality of life OR health status OR side effects OR long-term effects OR symptoms OR radiotherapy OR chemotherapy OR surgery OR anxiety OR sexuality OR infertility OR body image OR body mass index, weight change OR information OR treatment satisfaction OR insurance OR future perspective/uncertainty).

At this point, we brought forward operationalization of items (phase II) usually done after phase I to conduct assessments of specific item text already in patients and experts already at this stage. QOL issues collected so far were operationalized into items using a response format and time frame compatible with the EORTC QLQ-C30. Pre-existing items from the EORTC Quality of Life Group Item Bank (covering all items from all development stages of EORTC modules) were used where possible [27]. The English items were then translated into the languages of participating centres to allow collection of patient and expert ratings. Translation was done according to the EORTC translation procedure guidelines [28].

Expert and patient ratings of each item were collected for the following criteria:

- Relevance was rated for each item on a four-point scale, ranging from “not at all relevant” (1 point) to “very much relevant” (4 points). Relevance refers to the frequency with which a problem or symptom occurs and the trouble it may cause.
- Priority for inclusion was rated for each item to identify those items that affect patients′ QOL most and that should definitely be included in the final questionnaire.
- Breadth of coverage was investigated by asking patients and experts to suggest any relevant issues not included in the item list that should be added.

Items were eligible for inclusion if the mean relevance scores of patients and of specialists (considered separately) were 2 or greater and at least 40% of patients and 40% of specialists gave priority for inclusion. Furthermore, items were excluded if more than 25% of the answers were missing (this criterion was not applied to conditional items). These selection criteria for relevance and priority are similar to those used in phase I of other EORTC module development studies [29–31].

Ethical approval was obtained from local ethics committees at centres contributing patients to phase I and/or phase III.

### Phase III: pre-testing of the module

The pre-testing of the module in a sample of TC patients from different countries and with different languages aimed to identify potential problems regarding wording, comprehensiveness and redundancy or duplications. Patients were encouraged to comment on each question (e.g. was it difficult to answer, annoying, confusing, upsetting or intrusive) and to provide additional concerns or relevant QOL issues not mentioned in the questionnaire.

Retention criteria for phase III related to patient comments. Items were retained if <10% of patients made *any* negative comments about an item and if <5% of patients made *the same* negative comment about an item (e.g. <5% of patients complained about an item being difficult).

In addition, descriptive statistics and preliminary psychometric characteristics for a provisional, content-based subscale structure were determined. Scores have been linearly transformed to a scale range of 0–100, as is common for EORTC scales.

## Results

### Phase I and II: generation of relevant QOL issues and operationalization

The literature search revealed 37 articles and 26 questionnaires providing QOL issues relevant to TC patients. Based on this literature search and expert discussion, we assembled an initial list of 20 QOL areas containing 69 issues of potential relevance to TC patients. This list was edited to remove overlap and redundancy and was assessed by means of a semi-structured interview with experts in the field at two EORTC Quality of Life Group Meetings in 2006. These experts came from various countries (Austria, Belgium, Brazil, Germany, Italy, the Netherlands, Norway, Poland and the UK). Based on this selection procedure, we reduced the number of QOL issues on the list to 37 (see Table 1).

**Table 1:** QOL issues identified in phase I

QOL area	Issue
Treatment-related symptoms (in particular, chemotherapy)	1. Hair loss
	2. Different taste
	3. Sense of taste and smell
Treatment-related symptoms (in particular, radiotherapy)	4. Abdominal pain
	5. Heartburn
	6. Bloated feeling
	7. Change in bowel habit
	8. Skin problems
	9. Irradiated skin discoloured
Peripheral neuropathy	10. Tingling or numbness fingers/toes
	11. Pale/cold fingers/toes
	12. Burning/pain in fingers/toes
Difficulty in hearing/tinnitus	13. Problems with hearing
Satisfaction with medical management	14. Satisfied with care received
Satisfaction with received information	15. Satisfied with information received
Future uncertainty	16. Uncertain about future
Loan/Insurance	17. Loan/insurance problems
	18. Problems with job
Anxiety of recurrence	19. Anxious about a recurrence
Anxiety of family disruption	20. Disruption of family life
Communication	21. Talk about disease with partner
Activity	22. Physically limited
Infertility	23. Ability to have children
Body image	24. Less masculine
	25. Look at yourself naked
	26. Dissatisfied with body
	27. Satisfied with testicular implant
Weight change	28. Lost weight
	29. Gained weight
Sexual activity	30. Interested in sex
	31. Sexually active
Sexual functioning	32. Felt uncomfortable being intimate
	33. Talk about sexuality
	34. Difficulty getting an erection
	35. Problems with ejaculation
Sexual enjoyment	36. Was sex enjoyable?
Satisfaction with the sexual relationship	37. Satisfied with sexual relationship

The 37 issues were operationalized into items with a response format and time frame compatible with the QLQ-C30 (as mentioned above, we conducted phase I and II concurrently). For 25 issues, there were items available from the EORTC item bank. New items were created to assess the other 12 issues. These items were translated into Dutch, German, Italian and Spanish and evaluated by patients and experts.

Expert ratings on relevance, priority and breadth of coverage were collected from 28 experts (11 urologists, 6 radiation oncologists, 3 psychologists, 2 medical oncologists, 2 physicians, 2 junior physicians, a nurse and an urologist in training). They were working at centres in Austria (10), the Netherlands (7), Italy (7), Canada (3) and England (1). Their average years of professional experience was 11.9 (range 1–35). Items were rated separately for patients receiving treatment and patients after treatment.

The patient group for item evaluation included 62 TC patients from Austria (*n* = 39), Canada (*n* = 12) and the Netherlands (*n* = 11), with a mean age of 39.8 (SD 10.9). Detailed sociodemographic and clinical data are shown in Table 2.

**Table 2:** Phase I and III: Sociodemographic and clinical data for the module development samples

		Phase I sample *n* = 62	Phase III sample *n* = 156
Country (language)	Australia (English)	–	20.5%
	Austria (German)	62.9%	47.4%
	Canada (English)	19.4%	–
	Italy (Italian)	–	22.4%
	the Netherlands (Dutch)	17.7%	–
	Spain (Spanish)	–	9.6%
Age (years)	Mean (SD)	39.8 (10.9)	36.8 (10.5)
	Range	21-63	18-66
Education	Compulsory school or less	10.9%	14.2%
	Apprenticeship or professional	34.5%	29.1%
	School	38.2%	22.8%
	A-level university degree	16.4%	33.9%
Marital status	Single	31.0%	29.3%
	Married/partnership	62.1%	65.3%
	Divorced/separated	5.2%	4.0%
	Widowed	1.7%	1.3%
Employment	Full-time	75.4%	86.7%
	Part-time	7.0%	1.7%
	In training	3.5%	5.0%
	Unemployed	7.0%	2.5%
	Other	7.0%	4.1%
Treatment phase	On treatment	18.6%	21.0%
	<1st year of aftercare	14.0%	35.0%
	>1st year of aftercare	67.4%	44.1%
Tumour stage	Local	73.6%	74.8%
	Advanced	26.4%	25.2%
Surgery	Yes	91.2%	91.0%
	No	8.8%	9.0%
Radiotherapy	Yes	36.8%	10.2%
	No	63.2%	89.8%
Chemotherapy	Yes	47.4%	60.5%
	No	52.6%	39.5%

Twenty-six of the 37 items met all inclusion criteria relating to priority, relevance and breadth of coverage. The remaining 11 items failed to meet one criterion, mainly patient-rated relevance.

Comments by patients were very rare and therefore did not have a substantial impact on item selection and item wording. There were several comments from specialists regarding the content/wording of items (for item numbers refer to Table 3). Several items were revised based on the data collected. For example:

**Table 3:** Phase I: patient and expert ratings of item relevance and priority for inclusion

	Phase II items	Relevance ratings (mean)	Priority for inclusion (frequency)		
		Patients	Specialists off-/on-treatment	Patients (%)	Specialists off-/on-treatment (%)
1.	Did you have hair loss?	2.58	2.45/2.69	75	65/71
2	^a^Did food and drink taste different from usual?	2.47	2.05/2.21	76	36/31
3	Have you had problems with your sense of taste or smell?	2.19	2.50/2.67	73	52/56
4.	Did you have abdominal pain?	2.06	2.55/2.88	63	69/76
5	Have you had heartburn?	2.04	2.45/2.94	53	65/71
6	^a^Did you have a bloated feeling in your abdomen?	1.90	2.25/2.60	57	58/71
7	^c^Did you experience change in bowel habit as a result of your disease or treatment?	2.34	2.36/3.00	65	69/76
8	Have you had skin problems (e.g. itchy, dry)?	2.02	2.55/2.94	65	64/65
9	^a^Is the skin discoloured around the area that was irradiated?	1.98	2.23/2.44	62	52/53
10	^b^Have you had tingling or numbness in your fingers or toes?	1.93	2.75/2.87	72	76/75
11	Have you had pale/cold fingers or toes?	2.00	2.38/2.60	68	60/69
12	^a^Did you have burning and/or pain in your fingers or toes?	1.68	2.43/2.67	58	56/69
13	^b^Did you have problems with hearing?	1.69	2.81/2.53	56	68/69
14	Were you satisfied with the care you received from your doctors?	3.56	3.27/3.56	92	73/94
15	Were you satisfied with the information you received about your illness?	3.37	2.91/3.75	84	76/100
16	Did you feel uncertain about the future?	2.68	2.91/3.13	83	65/82
17	^a^Did you have any loan/insurance problems?	2.11	1.82/1.88	58	46/29
18	Have you had any problems with your job because of your illness?	2.00	2.48/2.88	67	72/71
19	Have you been anxious about a possible recurrence of the disease?	2.91	3.50/3.31	94	96/82
20	Were you concerned about disruption of family life?	2.49	2.86/3.31	79	60/76
21	Can you talk about your disease with your partner or the person who is closest to you?	3.52	3.00/3.50	94	62/88
22	Have you been physically limited as a result of your disease or treatment?	2.41	3.09/3.31	90	85/100
23	Were you concerned about your ability to have children?	2.43	3.41/3.81	87	92/100
24	^b^Have you felt less masculine as a result of your disease or treatment?	1.90	3.14/3.38	71	65/82
25	^a^Did you find it difficult to look at yourself naked?	1.63	2.64/3.13	49	42/71
26	^a^Have you been dissatisfied with your body?	1.79	2.77/3.06	52	54/71
27	Answer the question only if you have a testicular implant: Are you satisfied with your testicular implant?	3.00	3.22/3.60	79	87/83
28	^c^Have you lost weight?	2.14	2.81/2.53	83	72/47
29	^c^Have you gained weight?	2.05	2.76/2.44	76	64/41
30	To what extent were you interested in sex?	3.02	2.95/3.19	88	84/81
31	To what extent were you sexually active? (with or without intercourse)	2.66	2.64/2.69	84	73/71
32	^a^Have you felt uncomfortable about being sexually intimate?	1.98	2.95/3.13	71	69/82
33	Can you talk about sexuality with your partner or the person who is closest to you?	3.28	2.91/3.31	88	69/76
34	Did you have difficulty getting or maintaining an erection?	2.17	3.23/3.13	84	85/71
35	Did you have problems with ejaculation (e.g. dry ejaculation)?	2.17	3.14/2.94	81	85/71
36	To what extent was sex enjoyable for you?	3.20	2.32/2.38	87	46/53
37	Has the sexual relationship with your partner been satisfying?	3.29	2.59/2.63	85	58/65

^a^Exclusion due to failing at least one retention criterion

^b^Inclusion despite failing retention criteria, due to expert comments

^c^Exclusion due to expert comments, despite fulfilling retention criteria

- Item 4 “Did you have abdominal pain?” was changed to “Did you have pain in your stomach area?” to increase understandability.
- Item 7 “Did you experience change in bowel habit as a result of your disease or treatment?” was deleted as this issue is already covered by the QLQ-C30 questions on diarrhoea and constipation.
- Item 10 “Have you had tingling or numbness in your fingers or toes?” was retained, although patients rated the relevance of this item slightly below two. However, high expert-rated relevance provided grounds for inclusion.
- Item 13 “Did you have problems with hearing?” also received a relevance rating below two from patients, but high relevance ratings from experts supported inclusion.
- Item 18 “Have you had any problems with your job because of your illness?”was amended to include education, since a high percentage of patients with TC are quite young and may be still studying. Furthermore, we changed the term “illness” to “disease or treatment” to be consistent with other items.
- Item 24 “Have you felt less masculine as a result of your disease or treatment?” was retained, despite narrowly failing to meet the patient relevance criterion, because otherwise the module would not have covered the area of body image at all. This item was rated as highly relevant by specialists.
- Items 28 and 29 concerning weight gain and weight loss were deleted for several reasons. Experts considered it more appropriate to measure weight change using body mass index; weight change over a short period such as 4 weeks may be negligible for patients off-treatment (note that a different time frame was used for these two items); and weight gain and weight loss can not be combined in a meaningful single scale.

### Phase III: pre-testing of the module

The provisional TC module (EORTC QLQ-TC26) derived from phase II was pre-tested in four countries. From December 2008 to May 2010, a prospective sample of 156 TC patients was recruited in Austria (*n* = 74), Italy (*n* = 35), Spain (*n* = 15) and Australia (*n* = 32). Mean patient age was 36.8 years (SD 10.5), and mean time since diagnosis was 12.7 months (SD 14.8). Advanced disease was diagnosed in 25.2% of patients. For further details on patient characteristics see Table 2.

From the 156 patients who had been interviewed and had completed the questionnaire, 122 patients (78.2%) made no comments, whereas 34 patients (21.8%) commented on at least on one item. In total, patients provided 26 item-specific and 12 general comments.

Four patients (2.6%) found at least one question difficult to understand or answer (item 6 on skin problems and item 7 on pale/cold fingers), and nine patients (5.8%) reported at least one question to be upsetting, annoying or intrusive (items concerning sexuality, future uncertainty and body image). General comments related to the time frame of 7 days being too short and that questionnaires specific to treatment phases would be preferable. One new issue was raised referring to the need for information on sperm banking. It was agreed that this issue is of high importance before the start of treatment, but no new item was added, since we considered this issue to be covered by item 10 on satisfaction with the information received. The two patients commenting on item 7 (pale/cold fingers) were found to be patients in aftercare who had not undergone chemotherapy. The items on future perspective were considered important as they cover an important psychological parameter commonly assessed in cancer patients.

Overall, patients made only a low number of comments indicating good acceptance and understandability of the items. All items fulfilled the retention criteria stated in the methods section.

### Preliminary subscale structure

We conducted a psychometric analysis for a preliminary content-based subscale structure of the TC module (results are shown in Table 4). Most scales showed moderate to good internal consistency, but for the scales Sexual Enjoyment and Sexual Problems, internal consistency was relatively low. Detailed analysis revealed that this was most likely due to strong floor/ceiling effects and therefore limited item variance. As content appeared to be homogeneous, we decided to keep these scales at this stage.

**Table 4:** Proposed subscale structure for the QLQ-TC26 after phase III, with internal consistency statistics (Cronbach’s alpha) and descriptive statistics (item numbers refer to Table 5)

			On treatment		Within first year of aftercare		>1st year of aftercare		Total sample	
	Item	Cronbach’s alpha	Mean	SD	Mean	SD	Mean	SD	Mean	SD
Treatment Side effects^a^	01-08	0.78	20.0	12.7	13.9	18.4	8.2	12.1	12.5	15.2
Treatment satisfaction^b^	09–10	0.85	94.8	11.9	91.8	19.9	79.5	27.6	88.2	22.6
Future perspective^b^	11–12	0.76	48.9	29.0	54.8	25.2	70.4	26.4	60.8	27.8
Job problems^a^	13–14	0.80	43.7	33.2	26.4	26.4	18.5	29.9	25.4	30.9
Family problems^a^	15	Single item	38.1	38.2	29.9	33.1	26.3	31.4	28.9	32.8
Infertility^a^	16	Single item	24.1	39.7	34.7	38.9	31.7	36.5	30.2	37.3
Communication^b^	17, 21	0.62	92.2	13.7	82.0	25.9	78.0	27.0	82.9	24.5
Body image problems^a^	18	Single item	16.1	27.6	12.2	17.6	19.4	28.0	15.5	24.5
Sexual activity^b^	19–20	0.79	56.0	33.1	65.6	25.0	64.8	28.6	64.2	28.0
Sexual problems^a^	22–23	0.36	11.3	23.9	24.8	29.6	15.5	22.1	20.9	26.5
Sexual enjoyment^b^	24–25	0.51	76.2	27.7	77.3	25.7	72.5	26.4	73.6	26.4
Testicular Implant Satisfaction^b^	26	Single item	75.0	50.0	93.3	14.9	60.0	34.7	69.4	35.5

^a^Symptom scale (high scores indicate high impairment)

^b^Functioning scale (low scores indicate high impairment)

**Table 5:** EORTC QLQ-TC26 items (Phase III version)

#	Item text
1.	Have you lost any hair?
2.	Have you had problems with your sense of taste or smell?
3.	Have you had pain in your stomach area?
4.	Have you had acid reflux?
5.	Have you had tingling or numbness in your fingers or toes?
6.	Have you had skin problems (e.g. itchy, dry)?
7.	Have you had pale/cold fingers or toes?
8.	Did you have problems with hearing?
9.	Were you satisfied with the medical care you received?
10.	Were you satisfied with the information you received about your disease or treatment?
11.	Did you feel uncertain about the future?
12.	Have you been anxious about a possible recurrence of the disease?
13.	Have you had any problems with your job or your education because of your disease or treatment?
14.	Have you been physically limited as a result of your disease or treatment?
15.	Were you concerned about disruption of family life?
16.	Were you concerned about your ability to have children?
17.	Can you talk about your disease with your partner or the person who is closest to you?
18.	Have you felt less masculine as a result of your disease or treatment?
19.	To what extent were you interested in sex?
20.	To what extent were you sexually active? (with or without intercourse)
21.	Can you talk about sexuality with your partner or the person who is closest to you?
	*Next questions only in the case of sexual activity:*
22.	Did you have difficulty getting or maintaining an erection?
23.	Did you have problems with ejaculation?
24.	To what extent was sex enjoyable for you?
25.	Has the sexual relationship with your partner been satisfying?
	*Answer this question only if you have a testicular implant:*
26.	Are you satisfied with your testicular implant?

Descriptive statistics for the proposed subscales are given in Table 4 separately for patients in different treatment phases.

## Discussion

The EORTC QLQ-TC26 has been developed to measure disease and treatment-related QOL issues relevant to TC patients that are not covered by the EORTC QLQ-C30. The new module is designed to be administered together with the EORTC QLQ-C30. Module development in phase I, II and III was based on extensive literature search, and ratings and comments from experts and patients and followed the rigorous validation procedures of the EORTC Quality of Life Group [26]. To enhance cross-cultural applicability, patients from various European countries, Australia and Canada were included in the development process.

Across all development phases, we found that patients provided only a relatively small number of comments regarding items, whereas expert feedback and ratings contributed considerably to decisions on in/exclusion of items in the final version of the questionnaire. The low number of comments from patients indicating high acceptance may be due to the fact that a high proportion of items were derived from the EORTC item bank and had consequently already undergone selection procedures within other EORTC module development studies. As expected, a few patients expressed problems answering items on sexuality. Such items are well known to be problematic as they are inherently intrusive to some degree. This is often reflected by low response rates in clinical studies and has been found in other questionnaire development studies [29, 32, 33]. However, sexual functioning and sexual problems are important issues for TC patients and expected to have a particularly high impact on patients’ QOL. Therefore, these items were kept in the questionnaire module.

For QOL issue generation in phase I, we screened not only existing questionnaires specific to TC patients or to sexuality (e.g. the QOL questionnaire from Fossa et al. [25], and the Prostate Cancer Sexual Scale [34]), but also generic questionnaires (e.g. FACT-G [35] and the GHQ-28 [36]). As expected, multi-phase item refinement resulted in exclusion of most issues from generic questionnaires, as these were either covered by the EORTC QLQ-C30 core questionnaire or were only of minor relevance to TC patients. Due to the extensive nature of the initial item list generated in phase I, no new issues were included in phase III.

The questionnaire was developed for use not only in patients currently receiving treatment, but also in long-term survivors, that is, patients five or more years after treatment. As the relevance of specific QOL issues, in particular treatment side effects, changes from time of diagnosis to aftercare, extensive consideration was given to whether or not to define treatment phase–specific scales. Despite the advantage of avoiding items of potentially limited relevance to the current situation of an individual patient, we decided against multiple versions of the questionnaire, as this would have complicated longitudinal assessments of QOL across the whole disease trajectory. Also, QOL data collected in phase III showed that TC survivors more than 1 year from treatment still reported treatment side effects.

Related to this, a limitation of our study was that a large proportion of patients were in aftercare and were not undergoing active treatment at the time of assessment. While this reflects a characteristic of the TC patient population, it limited collection of patient feedback concerning treatment-related issues. In particular, patients treated with radiotherapy were underrepresented compared to figures from the literature [2]. Also, future changes in treatment strategies (e.g. increasing use of robotic surgery) may lead to a change in QOL issues relevant to TC patients.

Preliminary analysis of scale structure was primarily based on content at this stage. For several scales, Cronbach’s Alpha as measure of unidimensionality indicated sufficient item homogeneity. However, the scales Sexual Enjoyment and Sexual Problems were found to have poor internal consistency. This might be due to the above-mentioned presence of floor/ceiling effects in our sample, but could also lead to splitting these scales into single items. According to EORTC module development guidelines, definitive scale structure will be determined through field testing in phase IV. For now, we recommend combining these four sexuality items into two scales, as this is strongly suggested by content.

In conclusion, pre-testing of the EORTC QLQ-TC26 has been completed successfully. The developed questionnaire module proved to be applicable for the assessment of TC-specific QOL issues. Currently, the QLQ-TC26 is available in English, Dutch, German, Italian and Spanish. The questionnaire module will be developed further in an international field study (phase IV) investigating dimensionality, re-test reliability, sensitivity to change as well as the convergent and discriminatory validity of the scales.

## Abbreviations

EORTC: European Organisation for Research and Treatment of Cancer
FACT-G: Functional assessment of cancer therapy—general
GHQ-28: General Health Questionnaire-28
QLQ-C30: Quality of Life Questionnaire-Core 30
QOL: Quality of life
SD: Standard deviation
TC: Testicular cancer
